# The Role of Affects and Emotional Styles in the Relationship Between Parents and Preschool Children †

**DOI:** 10.3390/children11111369

**Published:** 2024-11-12

**Authors:** Carolina Facci, Andrea Baroncelli, Enrica Ciucci

**Affiliations:** 1 Department of Education, Languages, Interculture, Literatures and Psychology, University of Florence, Via di San Salvi 12, Complesso di San Salvi Padiglione 26, 50135 Florence, Italy; enrica.ciucci@unifi.it; 2 Department of Philosophy, Social Sciences and Education, University of Perugia, Piazza Giuseppe Ermini 1, 06123 Perugia, Italy; andrea.baroncelli@unipg.it

**Keywords:** parenting, parental feelings, children’s emotions, developmental trajectories

## Abstract

Background/Objectives: Parent–child relationships represent a key factor for the quality of developmental trajectories and impact on children’s social and emotional competence. Therefore, research has advanced the role of parenting by showing the significance of differentiating between distinctive aspects of a parent’s behaviors. This study aims to investigate the role of the feelings experienced in parent–child relationships (e.g., warmth and negative feelings), considering the moderating role of the parental styles toward children’s emotions (e.g., coaching and dismissing). Methods: A total of 136 mothers (M = 38.09 years, SD = 4.51 anni, 48.5% high school degree) with a preschool child (age range 3–5 years) in Central Italy have been involved in a survey during the pandemic period. Results: Multiple regression analyses show that warmth and negative feelings are associated with positive parenting; however, the moderation effect of the dismissing style on both warmth and negative feelings emerged. Conclusions: Despite the characteristics of the data collection period, the results suggest the importance of considering the emotion-related dimensions between parents and their children as they seem to influence parenting behaviors.

## 1. Introduction

Children’s socio-emotional development relies on the interaction between individual and contextual aspects. Research suggests that children’s emotional health could be linked to the association between aspects related to the child’s characteristics (e.g., child temperament) and quality of parenting (e.g., positive and negative parenting) [1,2]. Particularly, research established that parents’ behaviors and emotional competence—principally their emotion socialization behaviors and their emotional regulation—model the process of children’s emotional socialization [3,4] and impact children’s social and emotional competence as well as their maladaptive behaviors (e.g., conduct problems and CU behaviors) [5,6,7]. The ability to express, understand, and regulate emotions is a crucial element in socio-emotional development, particularly in young children. Moreover, the ability to identify and appropriately respond to emotional cues during a social interaction shapes their relationships. Research supports the idea that children’s socio-emotional competence has its developmental roots in the preschool years, and patterns of parenting appear to play an important role during early childhood [8].

According to the developmental perspective, parenting has long been described considering parents’ behaviors. Different levels of responsiveness and demandingness describe an authoritative, authoritarian, permissive, or indulgent style [9]. Recently, parenting has been recognized as a multidimensional construct, studied in terms of parent–child interactions that involve affection and acceptance (i.e., warmth) and discipline and rejection (i.e., control) [10]. The parent–child relationship has the power to shape the emotional climate of the family because it helps children feel supported and emotionally safe (e.g., free to express emotions) and is essential for the emotional socialization process and their socio-emotional development [11]. Parents have long been considered the first and most important socializers of emotional and social competence in the lives of their children [3,12]. The influences of the parent’s responsiveness and behaviors on children’s emotional socialization constitute a focal issue in developmental psychology [11,13]. Parent–child dyadic mutuality (i.e., shared positive affect, responsiveness, and cooperation) is recognized as an important component of family socialization processes [14,15]. Parents’ discussion and expression of emotions or reactions to their child’s emotions could outline the emotional climate of the family [3,16], shaping the quality of parent–child interactions. Also, parental beliefs and feelings about emotions drive parents’ emotional socialization behaviors toward their children [17,18,19]. The parental emotional styles (i.e., coaching and dismissing), based on beliefs and feelings, in responses to children’s negative emotions have been identified as a key feature for children’s socio-emotional development, because of their relations with children’s developing socio-emotional skills and difficulties [11,18].

### 1.1. Parents’ Emotional Competence and Meta-Emotion Philosophy

The emotional competence of the parents, which can be defined as the multifaceted ability to be aware of one’s own and others’ emotions, is predictive of parent–child relationship quality and the child’s behavioral outcomes [12]. Parent’s own emotions have received increasing attention in developmental research considering the influence on parenting behaviors and their role in creating the affective environment in which the children have been raised, which has an impact on their emotional and behavioral adjustment [20,21]. In addition, the rigor of the evaluation of parents’ emotional experiences is also given increased attention [22]. Parents with problems in accepting their own emotions may have difficulties engaging in supporting their children’s emotional socialization behaviors. When parents experience high levels of negative emotions, they may feel overwhelmed or ‘flooded’, increasing the likelihood of withdrawal or suppression of negative emotions, resulting in dismissal, or punitive discipline [23,24]. Parental problems in recognizing, accepting, and regulating their emotions tend to decrease emotional expressiveness and supportive parenting [21,24]. When parents have impairments in emotional competence and are less accepting of their own emotions, they may be less likely to talk overall about feelings and emotions [25].

Gottman, Katz, and Hooven [17] have proposed that parents hold a meta-emotion philosophy that involves their thoughts and feelings about their own emotions and the emotions of their children, and this is connected to the process of how they socialize emotions with their children. In more recent years, Katz and colleagues [18] showed and updated the meta-emotion philosophy framework that sustains the importance of parental beliefs, feelings, and attitudes toward emotions in children’s socio-emotional development. The belief that emotions are to be validated implies that there is recognition and acceptance of emotions as developmental benefits and opportunities for children to learn. Instead, the belief that emotions are dangerous may suggest less awareness and acceptance of emotions as an occasion to be supportive [13,26,27]. Parents’ beliefs and feelings about emotion shape parents’ perception of their child’s emotional experiences and their thoughts about how to teach emotions to their children, which mainly results in two emotional styles: coaching and dismissing [16,18,26]. Parents who dismiss or disapprove of the expression of negative emotions (i.e., sadness and anger) teach their children that these emotions are problematic or dangerous and perform behaviors such as denying, ignoring, or minimizing children’s emotions. Further, parents who coach the expression of negative emotions are supportive of their children’s expressions of these emotions and adopt behaviors such as emotional scaffolding, praising, and validation [17,28]. Although emotion coaching and emotion dismissing may seem to be opposite emotional styles, observational studies with children in middle childhood suggest that parents who engaged in both coaching and dismissing of children’s negative emotions had children with the lowest emotional dysregulation [29].

According to the updated meta-emotion framework of Katz and colleagues [18], parental meta-emotion philosophy, which is expressed by means of coaching and/or dismissing styles toward their children’s emotions, could have an impact on parenting behaviors and be influenced by both parent and child emotional experiences. In a recent study, it has been suggested that parental emotions influenced parenting behaviors, but they did not necessarily determine them [30]; parents could be guided by their beliefs about emotions to engage in consistent parenting behaviors [19,27]. Parental emotional styles may be particularly sensitive to parents’ emotional experiences because these styles reflect parents’ meta-emotion philosophy within parent–child interactions [26], and, consequently, these styles reflect parents’ intentions to scaffold or avoid children’s emotional expression and exploration. Gottman and colleagues [17,26], already argued that parental emotional coaching style affects parents’ inhibition of negative affect toward their children and facilitates positive parenting. The research on parents’ meta-emotion philosophy has been generative, and the assessment of this construct has been improved [21,31]. This construct incorporates different aspects of parent–child interaction (e.g., emotional beliefs, parenting strategies, and children’s emotional experiences) that require to be distinguished in order to better understand the process of socialization of emotion. Thus, an in-depth understanding of the role and relevance of parental meta-emotion philosophy, operationalized as parental coaching and dismissing styles, and the distinction between different parental aspects related to their emotional experience (i.e., parental feelings and behaviors) is essential when considering research on the emotion socialization process of children.

### 1.2. The Current Study

Based on the above-reported literature, the influence of the emotional experience of the parents in the parent–child relationship emerges. The main aim of the present study is to explore different dimensions of parenting, considering emotion-related dimensions (i.e., feelings and emotional style) as well as parental behaviors (i.e., positive, inconsistent, and punitive parenting) in a sample of mothers of preschool children.

In so doing, the associations between emotion coaching and emotion dismissing styles and parental feelings (i.e., warmth and negative feelings) in acting positive, inconsistent, and punitive parenting have been explored. Particularly, the aim was to examine whether parents may change their parental behaviors in association with parental feelings at different levels of emotional styles. A moderated model was tested to examine the unique and interactive effects between emotional styles and feelings in their association with parental behaviors. We hypothesized that parents experiencing negative emotions toward their children will be more likely to engage in negative parental behaviors at higher levels of beliefs that emotions can be problematic or dangerous (i.e., high level of dismissing style) [23,24]. We further hypothesized that parental coaching (i.e., recognition, acceptance, and regulation of children’s negative emotions, considering them as an opportunity for closeness) will moderate the relationships between negative feelings and engaging in positive parenting behaviors [25,26].

## 2. Materials and Methods

### 2.1. Participants and Procedure

The sample (N = 163) was recruited from kindergartens in Central Italy during the period of October and November 2021. Participants were all mothers, and the children ranged in age from 3 to 5 years (56 children in the class of the “oldest”—5 years old; 35 in the class of the “medium”—4 years old; 68 in the class of the “youngest”—3 years old; 4 attending a mixed class), which for the Italian educational system is the age range at which children can attend kindergartens. The mothers ranged in age from 26 to 49 years (Mage = 38.09, Ds = 4.51, 48.5% high school degree); however, three mothers did not report their age. Across samples, most mothers were from Italian cultural backgrounds (91.87%). The three scholastic institutions involved in the research approved and collaborated with all procedures. Participants were presented with a description of the study that was developed in collaboration with scholastic institutions and teachers, followed by a request to complete an informed consent to participate. Mothers provided informed consent and then completed an internet survey about their child, with demographic information and details about the parents (e.g., school degree, family composition). A series of scales were presented in a Google Form survey session, with no time restriction to fill out the survey.

### 2.2. Measures

#### 2.2.1. Parenting Behaviors

In the present study, we used the Alabama Parenting Questionnaire (APQ; [32]) preschool version (APQ-Pr; [33]) in the Italian version [34], which is a 32-item self-report test. The measure considers three dimensions of parenting: the 7-item inconsistent parenting subscale, which measures a lack of follow-through with discipline (e.g., “You threaten to punish your child and then do not actually punish him/her”); the 5-item punitive parenting subscale, which assesses how often a parent engages in corporal or harsh discipline (e.g., “You spank your child with your hand when he/she has done something wrong”); and the 12-item positive parenting subscale, which comprises items describing positive reinforcement and parental involvement (e.g., “You let your child know when he/she is doing a good job with something”). The respondent estimates the frequency of occurrence of each behavior on a 5-point Likert scale: 1 = never, 2 = almost never, 3 = sometimes, 4 = often, and 5 = always. The internal consistencies for the current sample were acceptable for the positive parenting scale (α = 0.76) and modest for the inconsistent parenting scale (α = 0.67) and punitive parenting scale (α = 0.60).

#### 2.2.2. Parental Feelings

In the present study, we used the Parent Feelings Questionnaire (PFQ; [35]), which is a 24-item measure that assesses both positive and negative parental feelings toward their children, and it is widely used as a measure of parental warmth in preschool samples [12]. The psychometric properties of the measure have been explored in other work [6]. The measure includes statements about feelings that are rated on a 5-point Likert-type scale, ranging from 1 (completely agree) to 5 (completely disagree). The measure includes two subscales: the parental warmth subscale (α = 0.60), which comprises 15 items assessing positive parental feelings (e.g., “When I think about this child, it usually gives me warm feelings”); and the negativity subscale (α = 0.90), which comprises 16 items assessing negative parental feelings (e.g., “Sometimes I am not happy about my relationship with this child”).

#### 2.2.3. Maternal Emotional Style

To assess the maternal emotional coaching (EC) and emotional dismissing (ED) styles, the Maternal Emotional Styles Questionnaire—MESQ, developed by Legacé Séguin and Coplan [36] in the Italian translation [37], was used. The psychometric properties of the measure have been explored in other work [6]. This instrument assesses the maternal emotional behaviors produced in response to children’s emotional displays. The 14 items represented a combination of fear, anger, and sadness emotions across the two emotional styles. Mothers are asked to express their level of agreement with each of the 14 items on a 5-point Likert scale ranging from 1 (strongly disagree) to 5 (strongly agree). Seven items describe EC (score range: 7–35; sample item: “When my child is angry, I take some time to experience this feeling with him/her”) and Cronbach’s alpha was 0.66 for this subscale; seven items describe ED (score range: 7–35; sample item: “When my child is angry, my goal is to make him/her stop”) and Cronbach’s alpha for this subscale was 0.81.

### 2.3. Data Analyses

All the analyses were conducted using SPSS version 28 [38]. First, bivariate correlations were used to examine the relations between all the variables. Then, a moderated model that examines the unique and interactive effects between emotional styles and feelings in their association with parental behaviors was performed. Hierarchical regression analyses explored the contributions of maternal feelings about children (i.e., each subscale of the PFQ) along with the potential moderating role of maternal emotional styles (i.e., both the emerged factors of the MESQ) to specific parental behaviors (i.e., positive, inconsistent, and punitive parenting). Although the directionality of the associations between the variables cannot be established in a cross-sectional study, the application of a regression approach requires assumptions about which variables to consider as independent and which to consider as criterion variables. The present analyses assumed that maternal feelings could influence maternal behaviors, and this is moderated by maternal emotional styles. Consequently, measures of feelings and behaviors were entered along with all scales of MESQ in Step 1, followed by the interaction terms between the two scales of MESQ and the two scales of feelings in Step 2. The form of results indicating significant interactions was explored using post hoc probing procedures indicated by Holmbeck [39].

## 3. Results

The distribution of study variables and bivariate correlations (Pearson’s r) among the study variables are provided in Table 1.

In this community sample, the distribution of the scales did not deviate significantly from normality. As expected, there were significant correlations between the three scales of parental behaviors and warmth and negativity. The coaching style was significantly positively correlated with positive parenting (*r* = 0.16, *p* < 0.05) and warmth (*r* = 0.28, *p* < 0.001). In addition, the dismissing style was positively correlated with all the parenting behaviors and negatively with negativity (*r* = −0.18, *p* < 0.05).

The results of the multiple regression analyses, testing the unique and interactive effects between emotional styles and feelings in their association with parental behaviors, are reported in Table 2.

**Figure 1:**
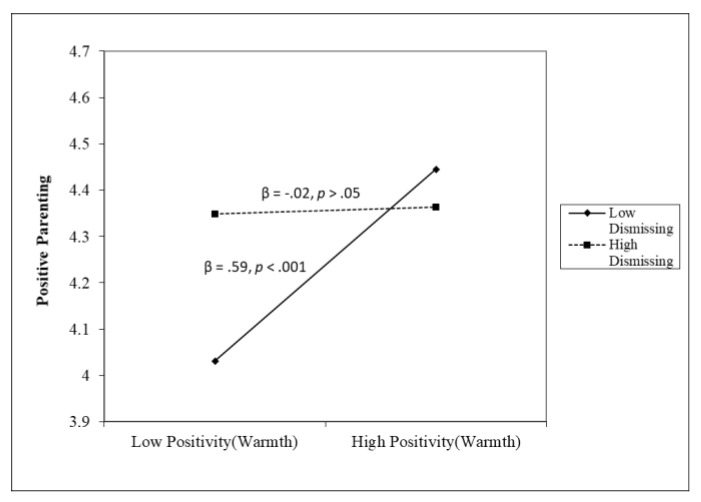
The moderating role of dismissing in the association between warmth and positive parenting.

**Figure 2:**
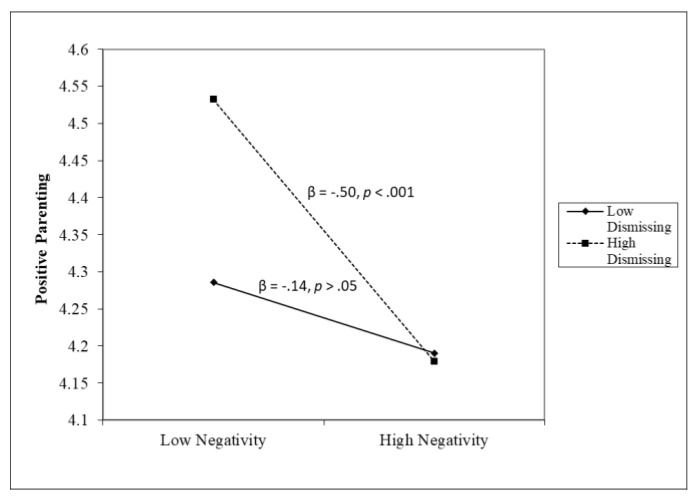
The moderating role of dismissing in the association between negativity and positive parenting.

Multiple regression analyses showed that warmth (β = 0.30, *p* < 0.01) and negativity (β = −0.32, *p* < 0.01) are associated with positive parenting; however, the moderation effect of dismissing style on warmth (β = −0.25, *p* < 0.01) and negativity (β = −0.17, *p* < 0.05) emerged. The form of the interaction provided in Figure 1 indicates that higher levels of positive feelings were related to higher positive parenting with low dismissing style (β = 0.59, *p* < 0.001) but not with high dismissing style (β = −0.02, *p* > 0.05). The form of the interaction provided in Figure 2 indicates that higher levels of negativity were related to lower positive parenting with a high dismissing style (β = −0.50, *p* < 0.001) but not with a low dismissing style (β = −0.14, *p* > 0.05). Moreover, multiple regression analyses showed that negativity (β = 0.29, *p* < 0.05) and dismissing style (β = 0.44, *p* < 0.001) are associated with inconsistent parenting; the dismissing style emerged positively associated with inconsistent parenting (β = 0.44, *p* < 0.001), and no moderation effect emerged between feelings and maternal emotional styles (F(10,162) = 5.256; *p* < 0.001; ΔR^2^ = 0.01, *p* = 0.88, R^2^ = 0.21). Moderate multiple regression analyses showed that warmth (β = −0.34, *p* < 0.01) and negativity (β = 0.33, *p* < 0.001) are associated with punitive parenting; the dismissing style emerged positively, even moderately, associated with punitive parenting (β = 0.18, *p* < 0.05), and no moderation effect emerged (F(10,162) = 6.334; *p* < 0.001; ΔR^2^ = 0.005, *p* = 0.56, R^2^ = 0.25).

## 4. Discussion

The current study investigated the role of emotion-related dimensions in engaging parental behaviors. Particularly, it explored the associations between parental emotional styles (e.g., coaching and dismissing styles) and feelings in acting parenting behaviors.

As expected, we found an association between the maternal emotional styles and the other parenting variables. Particularly, we found positive associations among warmth feelings, positive parenting scale, and coaching style. A negative association emerged between dismissing style and negative maternal feelings. We also found a positive association between the dismissing style and all the parenting behaviors (i.e., positive, inconsistent, and punitive parenting). Maternal warmth is strongly associated with parenting behaviors; positively with positive parenting but negatively with inconsistent and punitive parenting. Negative feelings are positively associated with inconsistent and punitive parenting but negatively associated with positive parenting. The result that maternal emotional style interacts with parenting behaviors and feelings is in line with the Parental Meta-Emotion Philosophy (PMEP) theory [18], which hypothesized that emotional socialization behaviors are guided by parents’ own emotion-related beliefs and feelings. Particularly, parents with a coaching style are responsive to their children’s emotions, support them to problem-solve, and are aware of the positive feelings about the relationship with the children. Surprisingly, a parent with a dismissing style who tends to reject or dismiss children’s emotions expresses fewer negative feelings and performs both positive and negative parenting behaviors. It is known that parental behaviors can be influenced by differences in children’s emotionality. Parents may experience distress and alter their views on how to teach their children about emotions [16,17,18] and how to relate based on their child’s emotionality. Children expressing negative emotions may make it harder for parents to engage in supportive emotion socialization behaviors. A parent who engages in a dismissing style is a parent who is uncomfortable with the expression of emotions, above all the negative ones. It may be speculated that mothers higher in dismissing style consider the child’s negative emotions as interpersonal challenges that put them out of their control and not as situations during which they perform their supportive parental role with their child. However, it has been shown that dismissing and coaching may not be the opposite style [29], and parents can adopt the dismissing style in the specific situation, engaging in both positive and negative parenting behaviors. There is evidence, even mixed, that parents’ emotions influence parenting behaviors [3,11]. It is suggested that parents with more understanding of their emotions are better able to apply supportive parenting behaviors [21,24]; various feelings may make it harder for parents to engage in positive parenting and a supportive emotional style [4]. Moreover, recognition and acceptance of negative feelings could be a protective factor from engaging in negative parenting behaviors [20].

A moderated model was tested to examine the unique and interactive effects between emotional styles and feelings in their association with parenting behaviors. We combined maternal feelings and maternal emotional styles into a regression model to highlight their role in parenting behaviors. The tested moderation model led us to make an in-depth exploration of the connection between the emotion-related parental dimensions. We expected to find the moderating role of both emotional styles on the association between negative feelings and parenting behaviors [23,24,28]. Contrary to our hypotheses, we found the moderating role of parental emotional styles only on positive parenting behaviors but not on negative ones. Our results suggested only the moderating role of maternal emotion dismissing style on the relation between both warmth and negative feelings on positive parenting; higher levels of warmth were related to higher positive parenting with low dismissing style. Warmth feelings could increase the probability of implementing positive parenting in parents with a low emotion dismissing approach to children’s emotions. Higher levels of negative feelings were related to lower positive parenting with a high-dismissing style. Negative feelings seemed to reduce the probability of implementing positive parenting in mothers with a high emotion dismissing style. It appears that warmth feelings are a protective factor for mothers with a low emotion dismissing style, and negative feelings are a risk factor for mothers with a high dismissing style. Mothers who believe that emotions can be problematic or dangerous for children (i.e., high emotional dismissing style) may hide or mask their own emotions in attempts to shield children from observing their emotional experiences. Therefore, the effort to dismiss emotions could create difficulties in engaging in positive parenting. At the same time, mothers who do not value emotions as challenging or risky (low dismissing style) may thus be more supportive and emotionally accepting toward the child’s emotions and experience positive feelings toward the child. We may suppose that emotion-dismissing parents are not less effectively involved than emotion-coaching parents; they may simply base their behaviors on an attitude that minimizes the role of feelings. Furthermore, parents may react positively or negatively to certain emotions depending on the nature of the emotion (e.g., sadness vs. anger). Thus, rather than simply sticking to a set of beliefs around emotions, parents may choose to behave with involvement and responsiveness in ways that are situationally and personally relevant to them and to their children.

Our results should be read in the context of some limitations. First, the cross-sectional nature of this study prevents us from making causal inferences. Future research should use longitudinal designs to explore the dynamics or causal relations among these variables. Second, both the size and representativeness of our sample limit the generalizability of results. The sample used in this study was primarily Italian, from central regions, and was of all mothers; these characteristics limit results interpretation to specific cultural and gender backgrounds. Different cultural perspectives may affect how parents think about emotions, specifically about children’s emotions [2,13]. Moreover, some studies have gone beyond the mother-child dyad and explored the importance of father-child dynamics; future research should explore the emotional style of fathers that could influence in different ways the children’s adjustments [2,19,40]. Another important aspect that could be considered in future research could be the income of the families that may be associated with a risk factor, such as stress, that can affect the quality and quantity of parental emotion styles [41]. Third, the data were collected only through parent report measures, and this may affect the risk of biasing the subjectivity of the results. Thus, future analyses could combine different measures, such as observational measures of the parent–child interactions.

## 5. Conclusions

The results of this study suggest the importance of considering the emotional dimensions of the parents toward their children, both in terms of feelings and emotional styles, as they would seem to influence parenting behaviors. Overall, our results suggest that during childhood, the parents’ belief about negative emotions to be denied influences their parenting positive behaviors, experiencing both positive and negative feelings. Parents controlling their negative feelings could make it harder to engage in positive parenting, but positive feelings protect them from not being supportive of the negative emotions of their children. Results from the present study should be interpreted considering the period of data collection (October–November 2020). The pandemic period could have influenced the family dynamics since a large body of research has identified a negative impact on individuals’ emotional health, both adults and children [42,43].

In conclusion, parents who are responsive and warm typically display specific types of parenting behaviors and have certain beliefs associated with emotions that lead to coaching and dismissing styles [3,25,26]. Research needs to examine how different parent–child interactions, particularly emotion-related dimensions, act together to outline parents’ behaviors. The results should be used to implement educational programs or training interventions for parents. Such programs should encourage both the level of emotional expressiveness and parents’ own emotion regulation skills [44].

## Figures and Tables

**Table 1:** Descriptive statistics and correlations (Pearson’s r) among study variables.

Variable	M	SD	Skew.	Kurt.	1	2	3	4	5	6	7
1 APQ—Positive Parenting	4.53	0.35	−0.92	0.57	/	−.01	−.04	.33 ***	−.35 ***	.16 *	.20 *
2 APQ—Inconsistent Parenting	2.55	0.71	0.11	−0.31		/	.16 *	−.20 **	.22 **	−.10	.39 ***
3 APQ—Punitive Parenting	2.17	0.63	0.60	0.52			/	−.43 ***	.34 ***	−.11	.16 *
4 PFQ—Warmth	4.68	0.39	−1.90	4.02				/	−.34 ***	.28 ***	−.10
5 PFQ—Negativity	2.31	0.80	0.40	−0.61					/	−.10	−.18 *
6 MESQ—Coaching	4.43	0.58	−1.19	1.50						/	−.06
7 MESQ—Dismissing	3.30	0.82	0.03	−0.67							/

Notes. APQ: Alabama Parenting Questionnaire. PFQ: Parent Feelings Questionnaire, MESQ: Maternal Emotional Style Questionnaire. * *p* < 0.05, ** *p* < 0.01, *** *p* < 0.001.

**Table 2:** Regression analyses testing the main and interactive effects of parental feelings and parental emotion socialization style on parenting behaviors.

	APQ- Positive	APQ- Inconsistent	APQ- Punitive	PFQ- Warmth	PFQ- Negativity	Coaching	Dismissing	F	R^2^
**APQ-** **Positive**	-	.02	.16 *	.30 ** (a)	−.32 ** (b)	.11	.17 *	(10,162) = 6.788 ***	.26
**APQ-** **Inconsistent**	.02	-	−.05	−.10	.29 ***	−.07	.44 ***	(10,162) = 5.256 ***	.21
**APQ-** **Punitive**	.16 *	−.04	-	−.34 ***	.32 ***	−.04	.18 *	(10,162) = 6.334 ***	.25

Notes. APQ: Alabama Parenting Questionnaire. PFQ: Parent Feelings Questionnaire. * *p* < 0.05, ** *p* < 0.01, *** *p* < 0.001. (a) There was a significant two-way interaction effect with dismissing in the association between warmth and positive parenting (β = −0.25, *p* < 0.01; F(10,162 = 6.788), *p* < 0.001; ΔR^2^ = 0.05, *p* < 0.01, R^2^ = 0.26), indicating that higher levels of warmth were related to higher positive parenting with low dismissing style (β = 0.59, *p* < 0.001) but not with high dismissing style (β = −0.02, *p* > 0.05). (b) There was a significant two-way interaction effect with dismissing in the association between negativity and positive parenting (β = −0.17, *p* < 0.05, F(10,162 = 6.788), *p* < 0.001, ΔR^2^ = 0.05, *p* < 0.01, R^2^ = 0.26), indicating that higher levels of negativity were related to lower positive parenting with high dismissing style (β = −0.50, *p* < 0.001) but not with low dismissing style (β = −0.14, *p* > 0.05).

## Data Availability

There are no unpublished data available. The corresponding author can be contacted regarding this matter.

## References

[1] Danzig A.P., Dyson M.W., Olino T.M., Laptook R.S., Klein D.N. (2015). Positive Parenting Interacts with Child Temperament and Negative Parenting to Predict Children’s Socially Appropriate Behavior. J. Soc. Clin. Psychol..

[2] Rothenberg W.A., Ali S., Rohner R.P., Lansford J.E., Britner P.A., Di Giunta L., Dodge K.A., Malone P.S., Oburu P., Pastorelli C. (2022). Effects of Parental Acceptance-Rejection on Children’s Internalizing and Externalizing Behaviors: A Longitudinal, Multicultural Study. J. Child Fam. Stud..

[3] Morris A.S., Silk J.S., Steinberg L., Myers S.S., Robinson L.R. (2007). The Role of the Family Context in the Development of Emotion Regulation. Soc. Dev..

[4] Rutherford H.J., Wallace N.S., Laurent H.K., Mayes L.C. (2015). Emotion Regulation in Parenthood. Dev. Rev..

[5] Duncombe M.E., Havighurst S.S., Holland K.A., Frankling E.J. (2012). The contribution of parenting practices and parent emotion factors in children at risk for disruptive behavior disorders. Child Psychiatry Hum. Dev..

[6] Facci C., Baroncelli A., Frick P.J., Ciucci E. (2024). Dimensions of Parenting and Children’s Conduct Problems: The Importance of Considering Children’s Callous-Unemotional Traits. Int. J. Environ. Res. Public Health.

[7] Kochanska G., Boldt L.J., Kim S., Yoon J.E., Philibert R.A. (2015). Developmental interplay between children’s biobehavioral risk and the parenting environment from toddler to early school age: Prediction of socialization outcomes in preadolescence. Dev. Psychopathol..

[8] Kochanska G., Boldt L.J., Goffin K.C. (2019). Early relational experience: A foundation for the unfolding dynamics of parent-child socialization. Child Dev. Perspect..

[9] Maccoby E.E., Martin J.A., Mussen P., Hetherington E. (1983). Socialization in the context of the family: Parent-child interaction. Handbook of Child Psychology: Vol. IV. Socialization, Personality, and Social Development.

[10] Deater-Deckard K., Lansford J.E., Malone P.S., Alampay L.P., Sorbring E., Bacchini D., Bombi A.S., Bornstein M.H., Chang L., Di Giunta L. (2011). The association between parental warmth and control in thirteen cultural groups. J. Fam. Psychol..

[11] Johnson A.M., Hawes D.J., Eisenberg N., Kohlhoff J., Dudeney J. (2017). Emotion socialization and child conduct problems: A comprehensive review and meta-analysis. Clin. Psychol. Rev..

[12] Denham S.A., Wyatt T.M., Bassett H.H., Echeverria D., Knox S.S. (2009). Assessing social-emotional development in children from a longitudinal perspective. J. Epidemiol. Community Health.

[13] Parker A.E., Halberstadt A.G., Dunsmore J.C., Townley G., Bryant A., Thompson J.A., Beale K.S. (2012). Emotions are a window into one’s heart”: A qualitative analysis of parental beliefs about children’s emotions across three ethnic groups. Monogr. Soc. Res. Child. Dev..

[14] Kim S., Boldt L.J., Kochanska G. (2015). From parent-child mutuality to security to socialization outcomes: Developmental cascade toward positive adaptation in preadolescence. Attach. Hum. Dev..

[15] Deater-Deckard K., O’Connor T.G. (2000). Parent-child mutuality in early childhood: Two behavioral genetic studies. Dev. Psychol..

[16] Eisenberg N., Cumberland A., Spinrad T.L. (1998). Parental Socialization of Emotion. Psychol. Inq..

[17] Gottman J.M., Katz L.F., Hooven C. (1997). Meta-Emotion: How Families Communicate Emotionally.

[18] Katz L.F., Maliken A.C., Stettler N.M. (2012). Parental meta—Emotion philosophy: A review of research and theoretical framework. Child Dev. Perspect..

[19] Dunsmore J.C., Her P., Halberstadt A.G., Perez-Rivera M.B. (2009). Parents’ Beliefs about Emotions and Children’s Recognition of Parents’ Emotions. J. Nonverbal Behav..

[20] Dunsmore J.C., Booker J.A., Ollendick T.H. (2013). Parental Emotion Coaching and Child Emotion Regulation as Protective Factors for Children with Oppositional Defiant Disorder. Soc. Dev..

[21] Meyer S., Raikes H.A., Virmani E.A., Waters S., Thompson R.A. (2014). Parent Emotion Representations and the Socialization of Emotion Regulation in the Family. Int. J. Behav. Dev..

[22] Zhang X., Beatty A., Abela K., Melo M.F., Kenny M., Atkinson L., Gonzalez A. (2023). Assessing parental emotion regulation in the context of parenting: A systematic review. Dev. Rev..

[23] Lorber M.F., Mitnick D.M., Slep A.M. (2016). Parents’ experience of flooding in discipline encounters: Associations with discipline and interplay with related factors. J. Fam. Psychol..

[24] Mence M., Hawes D.J., Wedgwood L., Morgan S., Barnett B., Kohlhoff J., Hunt C. (2014). Emotional flooding and hostile discipline in the families of toddlers with disruptive behavior problems. J. Fam. Psychol..

[25] Halberstadt A.G., Eaton K.L. (2002). A Meta-Analysis of Family Expressiveness and Children’s Emotion Expressiveness and Understanding. Marriage Fam. Rev..

[26] Gottman J.M., Katz L.F., Hooven C. (1996). Parental meta-emotion philosophy and the emotional life of families: Theoretical models and preliminary data. J. Fam. Psychol..

[27] Lozada F.T., Halberstadt A.G., Craig A.B., Dennis P.A., Dunsmore J.C. (2016). Parents’ Beliefs about Children’s Emotions and Parents’ Emotion-Related Conversations with Their Children. J. Child Fam. Stud..

[28] Cleary R., Katz L.F. (2008). Family-level emotion socialization and children’s comfort with emotional expressivity. Fam. Psychol..

[29] Lunkenheimer E.S., Shields A.M., Cortina K.S. (2007). Parental emotion coaching and dismissing in family interaction. Soc. Dev..

[30] Hajal N.J., Paley B. (2020). Parental emotion and emotion regulation: A critical target of study for research and intervention to promote child emotion socialization. Dev. Psychol..

[31] Paterson A.D., Babb K.A., Camodeca A., Goodwin J., Hakim-Larson J., Voelker S., Gragg M. (2012). Emotion-Related Parenting Styles (ERPS): A Short Form for Measuring Parental Meta-Emotion Philosophy. Early Educ. Dev..

[32] Shelton K.K., Frick P.J., Wootton J. (1996). Assessment of parenting practices in families of elementary school-age children. J. Clin. Child Psychol..

[33] Clerkin S.M., Marks D.J., Policaro K.L., Halperin J.M. (2007). Psychometric properties of the Alabama parenting questionnaire-preschool revision. J. Clin. Child Adolesc. Psychol..

[34] Benedetto L., Ingrassia E.M. (2014). L’Alabama Parenting Questionnaire per la fascia prescolare (APQ-Pr). Contributo all’adattamento italiano. Disturbi Attenzione Iperattività.

[35] Deater-Deckard K. (1996). Parent Feelings Questionnaire. J. Abnorm. Psychol..

[36] Lagaceé-Séguin D.G., Coplan R.J. (2005). Maternal emotional styles and child social adjustment: Assessment, correlates, outcomes and goodness of fit in early childhood. Soc. Dev..

[37] Ciucci E., Menesini E., Albanese O., Molina P. (2008). La comprensione delle emozioni nei bambini e lo stile emotivo materno. Lo Sviluppo Della Comprensione Delle Emozioni e la sua Valutazione.

[38] IBM Corp (2021). IBM SPSS Statistics for Windows, Version 28.0..

[39] Holmbeck G.N. (2002). Post-hoc Probing of Significant Moderational and Mediational Effects in Studies of Pediatric Populations. J. Pediatr. Psychol..

[40] Baker J.K., Fenning R.M., Crnic K.A. (2011). Emotion Socialization by Mothers and Fathers: Coherence among Behaviors and Associations with Parent Attitudes and Children’s Social Competence. Soc. Dev..

[41] Taraban L., Shaw D.S. (2018). Parenting in context: Revisiting Belsky’s classic process of parenting model in early childhood. Dev. Rev..

[42] Matiz A., Fabbro F., Paschetto A., Urgesi C., Ciucci E., Baroncelli A., Crescentini C. (2022). The Impact of the COVID-19 Pandemic on Affect, Fear, and Personality of Primary School Children Measured During the Second Wave of Infections in 2020. Front. Psychiatry.

[43] Facci C., Iacopino M., Baroncelli A., Ciucci E. The rise of online teaching and digital learning during the health emergency from COVID-19 and teachers’ working self-efficacy: An Italian perspective. Proceedings of the 3rd Symposium on Psychology-Based Technologies.

[44] Havighurst S.S., Wilson K., Harley A.E., Prior M.R., Kehoe C. (2010). Tuning in to kids: Improving emotion socialization practices in parents of preschool children—Findings from a community trial. J. Child. Psychol. Psychiatry.

